# Supplementary material for: The Systems Biology Research Tool: evolvable open-source software
Source: BMC Syst Biol. 2008 Jun 29;2:55. doi: 10.1186/1752-0509-2-55 (PMC2446383; doi:10.1186/1752-0509-2-55)
Supplement: Additional file 1 — SBRT Archive. An archive of the current version of the Systems Biology Research Tool. [file 1752-0509-2-55-S1.zip › sbrt-1.4.0/doc/developers_guide/api/sbrt/shell/io/MapOutputFile.html]

MapOutputFile


|  |  |  |
| --- | --- | --- |
| **Overview** \| **Package** \| **Class** \| **Use** \| **Tree** \| **Deprecated** \| **Index** \| **Help** | |  |
| **PREV CLASS**   **NEXT CLASS** | **FRAMES**    **NO FRAMES**     **All Classes** |  |
| SUMMARY: NESTED \| FIELD \| CONSTR \| METHOD | DETAIL: FIELD \| CONSTR \| METHOD |  |


---


## sbrt.shell.io Interface MapOutputFile<K,V>

**Type Parameters:**: `K` - the variable type.: `V` - the value type.

**All Superinterfaces:**: OutputFile<java.util.Map<? extends K,? extends V>>, ResultsManager<java.util.Map<? extends K,? extends V>>, SbrtFile

**All Known Subinterfaces:**: MapTextOutputFile<K,V>

**All Known Implementing Classes:**: AbstractMapOutputFile, ComoFile, RomoFile, SmoFile

---

``` public interface MapOutputFile<K,V> extends OutputFile<java.util.Map<? extends K,? extends V>> ```

This interface is used to represent output files that
maps can be written to.

**Author:**
:   This interface was written and documented by
    Jeremiah Wright while in the Wagner lab.

---

| **Method Summary** | |
| --- | --- |
| `void` | `addData(java.util.Map<? extends K,? extends V> map)`             Writes the appropriate values of the provided map to this file. |
| `java.util.Set<K>` | `getFilterKeys()`             Returns the set of keys whose values will be written to this output file when a map is added. |

**Methods inherited from interface sbrt.shell.io.SbrtFile**

`close, getCanonicalName, getOriginalName, isClosed`

**Methods inherited from interface sbrt.kernel.io.ResultsManager**

`addData, breakPoint, close, getName, isClosed`

**Method Detail**

### getFilterKeys

```
java.util.Set<K> getFilterKeys()
```

:   Returns the set of keys whose values will be
    written to this output file when a map is added.
    This set acts as a type of filter to control which
    values in a map are saved, and which are discarded.

    :   **Returns:**: the set of keys whose values will be written to this output file when a map is added.

---


### addData

```
void addData(java.util.Map<? extends K,? extends V> map)
```

:   Writes the appropriate values of the provided map
    to this file. The appropriate values are those of the
    keys in the set returned by `getFilterKeys()`.

    :   **Parameters:**: `map` - the map to be written to this file. **Throws:**: `java.lang.IllegalArgumentException` - if the provided map does not contain all the keys in the set returned by `getFilterKeys()`.: `java.lang.IllegalStateException` - if `close()` has already been called.


---


|  |  |  |
| --- | --- | --- |
| **Overview** \| **Package** \| **Class** \| **Use** \| **Tree** \| **Deprecated** \| **Index** \| **Help** | |  |
| **PREV CLASS**   **NEXT CLASS** | **FRAMES**    **NO FRAMES**     **All Classes** |  |
| SUMMARY: NESTED \| FIELD \| CONSTR \| METHOD | DETAIL: FIELD \| CONSTR \| METHOD |  |


---
